# Chewing the fat for good health: ACSM3 deficiency exacerbates metabolic syndrome

**DOI:** 10.1038/s44318-024-00037-0

**Published:** 2024-01-23

**Authors:** Juliana Cazarin, Brian J Altman

**Affiliations:** 1 Department of Biomedical Genetics, University of Rochester School of Medicine and Dentistry, Rochester, NY USA; 2 Wilmot Cancer Institute, University of Rochester Medical Center, Rochester, NY USA; 3 Member, Catalysts Program, The EMBO Journal, EMBO, Heidelberg, Germany

**Keywords:** Metabolism, Molecular Biology of Disease

## Abstract

Recent work identifies mitochondrial acyl-CoA synthetase ACSM3 as a guardian of hepatic lipid processing and metabolic health in mice and patients.

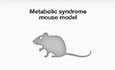

## Teaser

The origin of metabolic syndrome, a pathophysiological condition facilitating cardiovascular disease, diabetes, and other comorbidities, remains poorly understood. Recent work by Xiao et al (2023) identifies the mitochondrial enzyme ACSM3 as a determinant of lipid homeostasis and hepatic health in mice and patients. Additionally, the authors identified deleterious downstream p38-MAPK signalling as a targetable feature of ACSM3 deficiency that may help to ameliorate metabolic syndrome.

Metabolic syndrome (MetS) constitutes a cluster of pathophysiological conditions, including obesity, dyslipidemia, hypertension, dysregulated glucose homeostasis, and insulin resistance. This condition occurs in around 20–30% of the population worldwide and represents a major risk factor for cardiovascular disease, diabetes, and other comorbidities, being associated with higher mortality (Saklayen, 2018). MetS pathogenesis is multifactorial, arising from both genetic and acquired components such as energy expenditure, diet, and lifestyle, that mutually interact to drive the dysregulation of whole-body metabolic homeostasis (Lusis et al, 2008). A better understanding of the complex molecular mechanism underlying these metabolic alterations is key for the improvement of therapeutic interventions.

Aberrant lipid catabolism is an important component of metabolic syndrome and can result in dyslipidemia, manifested as increased circulating triglycerides and fatty liver, but the causes of this alteration in fatty acid processing remain unclear (Rada et al, 2020). Acyl-CoA synthetases play an essential role in mitochondrial lipid breakdown, catalyzing the conjugation of CoA (coenzyme A) to fatty acids and generating acyl-CoA metabolic intermediates. Acyl-CoA is then transported from the cytosol to the mitochondrial matrix and shuttled into lipid catabolic pathways such as β-oxidation. ACSM3 (acyl-CoA synthetase medium-chain family member 3) is an acyl-CoA synthetase that targets medium-chain fatty acids (C4-C14), and alterations in its expression have been previously associated with MetS. Junková et al (2021) compared two MetS rat models, and found that one of the models was more susceptible to developing high-fat diet-induced MetS with markedly elevated triglyceride levels (Junková et al, 2021). Interestingly, liver transcriptomic analysis of this rat model revealed a deficiency in lipid utilization, which correlated with the lack of liver *Acms3* expression observed. A potential role of ACSM3 in MetS is further supported by associations of ACSM3 polymorphisms with obesity, hypertriglyceridemia, and hypertension in patient cohorts (Iwai et al, 2002; Telgmann et al, 2007). However, these studies did not reveal any mechanistic insights into how lower ACSM3 may either cause or exacerbate MetS.

**Figure 1:**
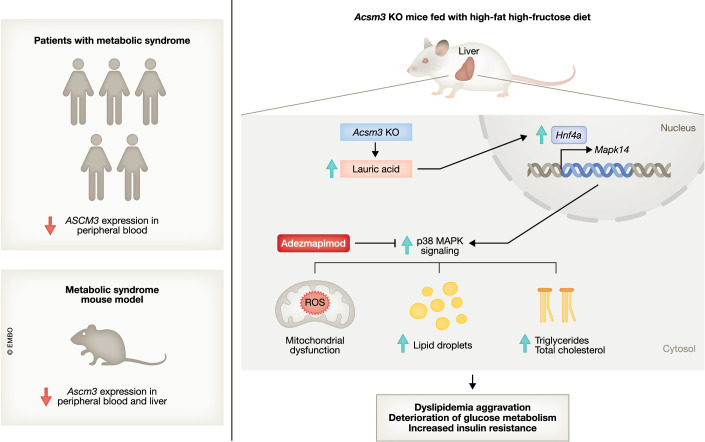
Role of ACSM3 in metabolic syndrome. (**Left Panel**) *Acsm3 is* downregulated in the blood of patients and mice with metabolic syndrome. In mice, diet-induced metabolic syndrome also represses hepatic *Acsm3* expression. (**Right Panel**) *Acsm3* deficiency results in lauric acid accumulation in the liver which activates *Hnf4α* and increases the transcription of *Mapk14* and the activation of the p38-MAPK pathway. The p38-MAPK signaling drives abnormal lipid metabolism, lipid accumulation, and mitochondrial disruption in the liver, aggravating dyslipidemia, insulin resistance, and dysregulation of glucose metabolism.

In the current mechanistic study, Xiao et al (2023) sought to better understand how ACSM3 expression relates to MetS in humans, and how its deficiency may worsen the manifestation of MetS (Fig. 1). First, the authors identified that ACSM3 gene expression was markedly downregulated in the peripheral blood of MetS patients compared with control individuals in a male patient cohort. This was further confirmed in a larger (*n* = 826), gender-balanced, and independent second cohort, corroborating a potential implication of ACSM3 in human MetS (Fig. 1, Left Panel). In mice, diet-induced metabolic syndrome also resulted in the repression of *Acsm3* gene expression in peripheral blood. ACSM3 is highly expressed in the mouse liver, and liver ACSM3 was dramatically repressed in both mRNA and protein levels in MetS mice (Xiao et al, 2023). It is well established that the liver plays a central role in glucose, lipid, and cholesterol metabolism and its dysfunction is frequently implicated in MetS (Lim et al, 2021). However, the causal implication of ACSM3 downregulation in liver metabolic dysfunction and the pathogenesis of MetS remained unclear.

To address this gap, Xiao et al (2023) generated *Acsm3* whole-body knockout male mice. In animals fed under a normal diet, systemic *Acsm3* depletion resulted in mild impairment in glucose homeostasis and decreased insulin sensitivity, with no alterations in both fasting glucose and insulin levels between groups. Serum and hepatic triglycerides were higher in the KO mice when compared with the wild-type, but no alterations in serum and hepatic total cholesterol, HDL, LDL, or non-esterified fatty acids (NEFA) were found between groups. Since the alteration in metabolic phenotype driven by *Acsm3* KO was mild in animals under a normal diet, the authors decided to investigate whether *Acsm3* KO was able to aggravate metabolic dysfunction in a MetS model driven by high fructose-high-fat (FF) diet. Overall, *Acsm3* KO mice presented a more severe MetS than wild-type animals, displaying impairment of glucose homeostasis, increased insulin resistance, and higher levels of serum and hepatic total cholesterol and triglycerides, NEFA, HDL, and LDL. Interestingly, liver-specific knockdown of *Acsm3* phenocopied the main metabolic alterations found in the systemic *Acsm3* KO, suggesting a central role of hepatic ACSM3 in MetS. In the liver, the increased hepatic accumulation of medium-chain FAs, along with significant increases in lipid deposition, were observed in the KO mice. Higher hepatocellular ballooning (swelling in size) and serum alanine aminotransferase (ALT) and aspartate aminotransferase (AST) were detected in the KO mice, which are indicative of hepatocyte degeneration and liver damage, respectively. This damage may center on the mitochondria: the liver of *Acsm3* KO displayed abnormal mitochondrial morphology, and hepatocytes from KO animals showed decreased oxygen consumption, decreased intracellular ATP levels, increased reactive oxygen species, and reduced mitochondrial membrane potential which is suggestive of mitochondrial dysfunction.

To further investigate the mechanisms underlying the hepatic dysfunction secondary to *Acsm3* KO, the authors performed a transcriptomic analysis of the liver from FF-fed mice and identified enrichment in pathways associated with p38-MAPK signaling. The p38-MAPK signaling pathway was a particularly important hit, as it has been previously implicated in both metabolic syndrome severity and mitochondrial dysfunction (Nikolic et al, 2020). The authors observed that p38 was significantly activated in the liver of FF-fed *Acsm3* KO mice, and followed this observation by treating primary hepatocytes from FF-fed *Acsm3* KO mice with the p38-MAPK inhibitor Adezmapimod (SB203580), a tool compound whose derivatives are being investigated for future clinical use (Machado et al, 2021). Adezmapimod completely rescued mitochondrial dysfunction, suggesting that this pathway could be driving the MetS aggravation observed in these animals. Corroborating this idea, the treatment of FF-fed *Acsm3* KO mice with Adezmapimod resulted in an overall improvement in glucose metabolism, reduction of insulin resistance, diminished hepatic lipid deposition, and ballooning degeneration. Together, these data demonstrate that ACMS3 suppression contributes to MetS aggravation in a p38-MAPK-dependent pathway. However, the mechanism by which Acsm3 loss promoted activation of p38 remained unclear.

The authors elegantly solved this puzzle by identifying lauric acid (C12), a medium-chain saturated FA, as a mediator of p38-MAPK activation in the liver. Lauric acid is highly accumulated in mice’s liver in response to *Acsm3* KO, and the exposure of primary hepatocytes to this FA activated the p38 cascade in an Hnf4α-dependent fashion. This suggested that liver cells lacking Acsm3 were unable to properly metabolize and degrade medium-chain fatty acids such as lauric acid. Diets enriched in medium-chain fatty acids have been previously demonstrated to induce the accumulation of liver triglycerides and cause hepatic insulin resistance (Turner et al, 2009), mirroring the hepatic metabolic phenotype observed in the *Acsm3* KO mice. In primary hepatocytes, lauric acid impaired hepatic insulin signaling through an HNF4α-mediated mechanism, indicating its role as a regulator of hepatic metabolism (Kamoshita et al, 2022). In agreement with these findings, the authors demonstrated that lauric acid increased Hnf4α activity, which in turn increases the transcription of *Hnf4a* itself and *Mapk14*, which encodes p38α. In conclusion, the authors demonstrate that suppression of *Acsm3* drives MetS by inducing hepatic mitochondrial dysfunction in a lauric acid-Hnf4α-p38-MAPK-dependent pathway (Fig. 1, Right Panel).

Overall, this work suggests that targeting the p38-MAPK pathway might be an effective approach to attenuate dyslipidemia and alterations in glucose homeostasis observed in MetS. Despite the fact that p38 inhibitors are not yet approved for clinical use, several compounds are under pre-clinical and clinical investigation (Machado et al, 2021). Interestingly, the authors of this work demonstrate that ACMS3 suppression in MetS patients and mice is detectable in blood. Thus, ACMS3 expression might be a good candidate as a blood-based biomarker to identify the MetS patients that will benefit from therapies targeting p38.
